# Supplementary material for: DNA from non-viable bacteria biases diversity estimates in the corals Acropora loripes and Pocillopora acuta
Source: Environ Microbiome. 2023 Dec 8;18:86. doi: 10.1186/s40793-023-00541-6 (PMC10704692; doi:10.1186/s40793-023-00541-6)
Supplement: Supplementary file 2 — Additional file 2. Supplementary Figures S1–S5. Supplementary Figure S1: Box plots showing 16S rRNA gene copies μl−1 of method validation samples from ddPCR. Supplementary Figure S2: Rarefaction curves for each of six coral species sampled. Supplementary Figure S3: Alpha diversity indices by coral genotype for A. kenti and P. daedalea. Supplementary Figure S4: Relative abundance of bacterial genera for A. millepora, A. kenti, P. daedalea, and P. lutea by genotype. Supplementary Figure S5: Relative abundance of Endozoicomonas and unknown Rhodanobacteraceae ASVs by coral genotype. [file 40793_2023_541_MOESM2_ESM.docx]

Supplementary Materials for

**DNA from non-viable bacteria biases diversity estimates in the corals *Acropora loripes* and *Pocillopora acuta***

Ashley M. Dungan*, Laura Geissler, Amanda S. Williams, Cecilie Ravn Gotze, Emily C. Flynn, Linda L. Blackall,

Madeleine J. H. van Oppen

*Corresponding author. Email: adungan31@gmail.com

**This file includes Figures S1-S5.**


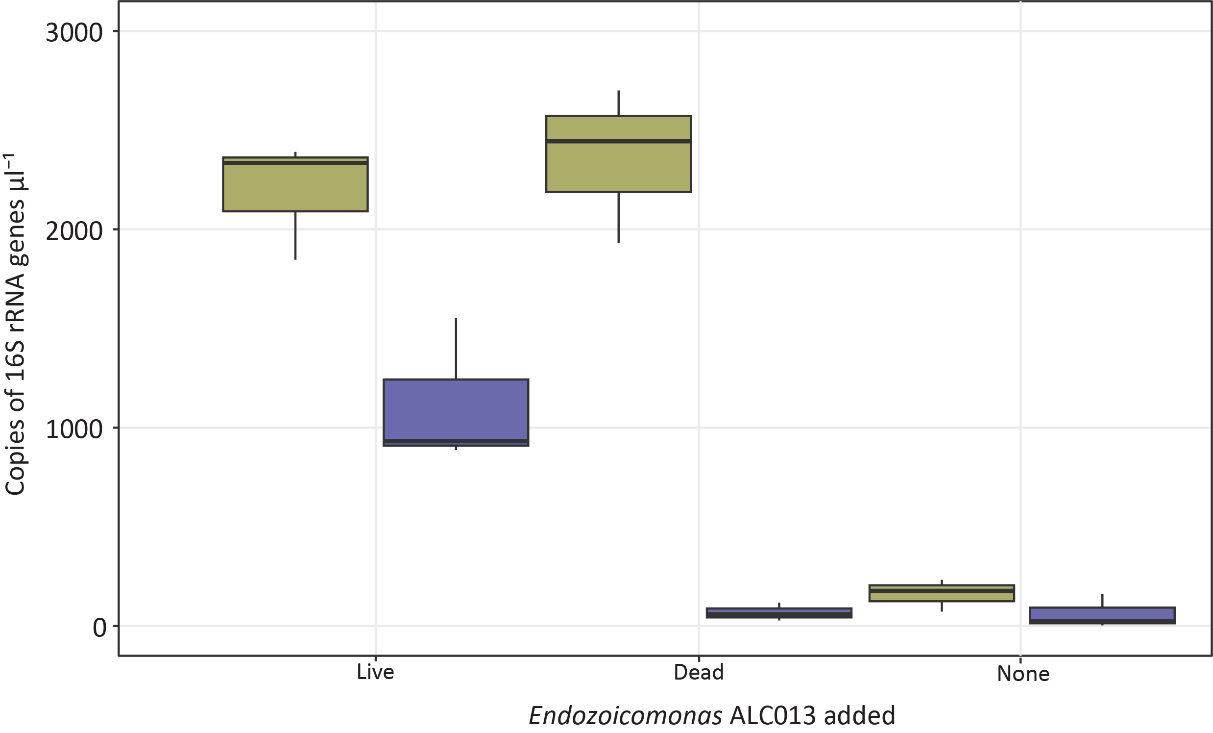


Figure S1: Box plots showing 16S rRNA gene copies µl^-1^ of samples from ddPCR for heat-killed anemone homogenate spiked with viable (Live) or heat-killed (Dead) Endozoicomonas sp. or unspiked (None) and either untreated (gold) or PMA-treated (purple). PMA treatment successfully precluded PCR when samples were spiked with heat-killed bacterial cells. For each plot, n=3.


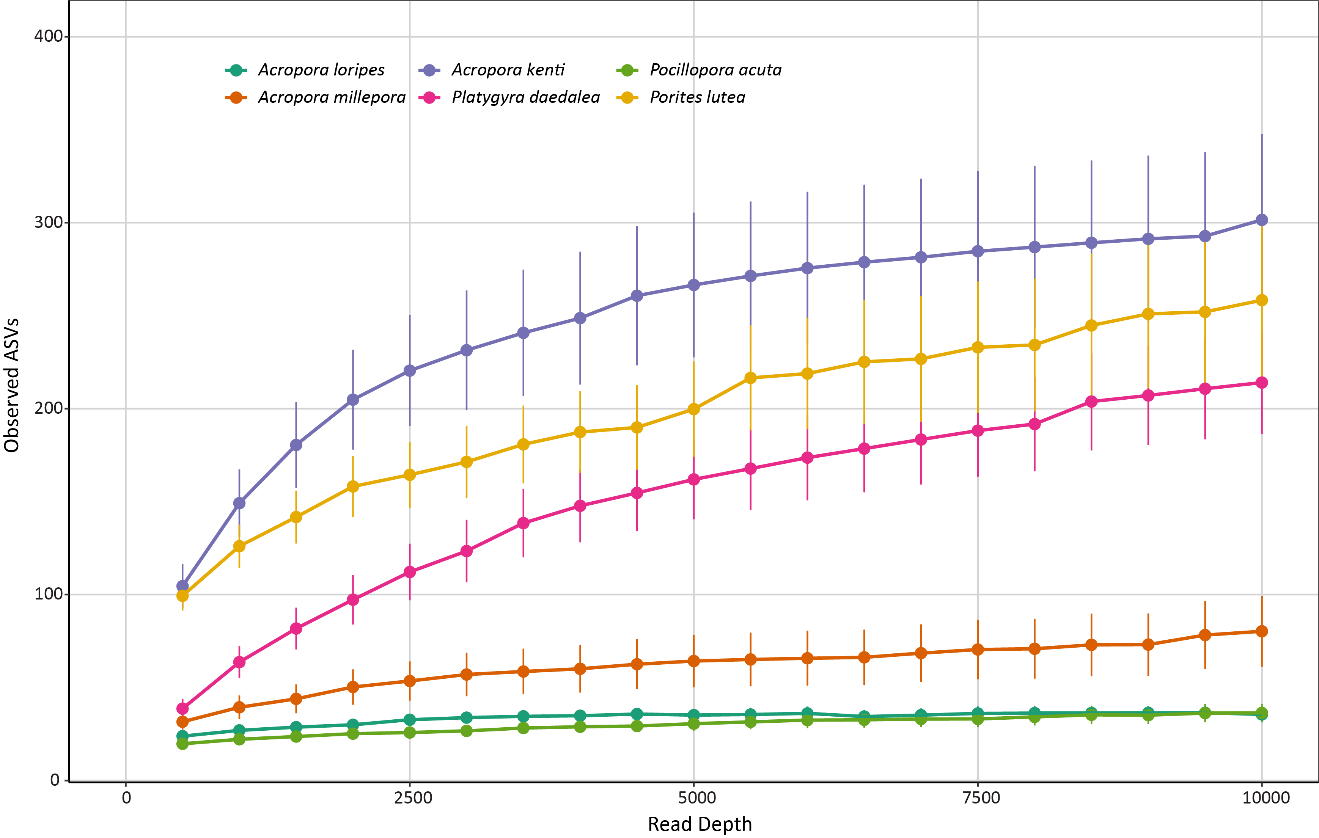


Figure S2: Rarefaction curve showing the relationship between sequencing depth and species richness (Observed ASVs) for each of six coral species sampled (from most to least diverse: Acropora kenti – purple; Porites lutea – yellow; Platygyra daedalea – pink; A. millepora – orange; A. loripes – dark green; Pocillopora acuta – light green). Rarefactions were computed in QIIME2 using the ‘alpha-rarefaction’ function with ten iterations at intervals of 500 up to 10000 reads sampled. Standard error for each point is shown with the error bars.


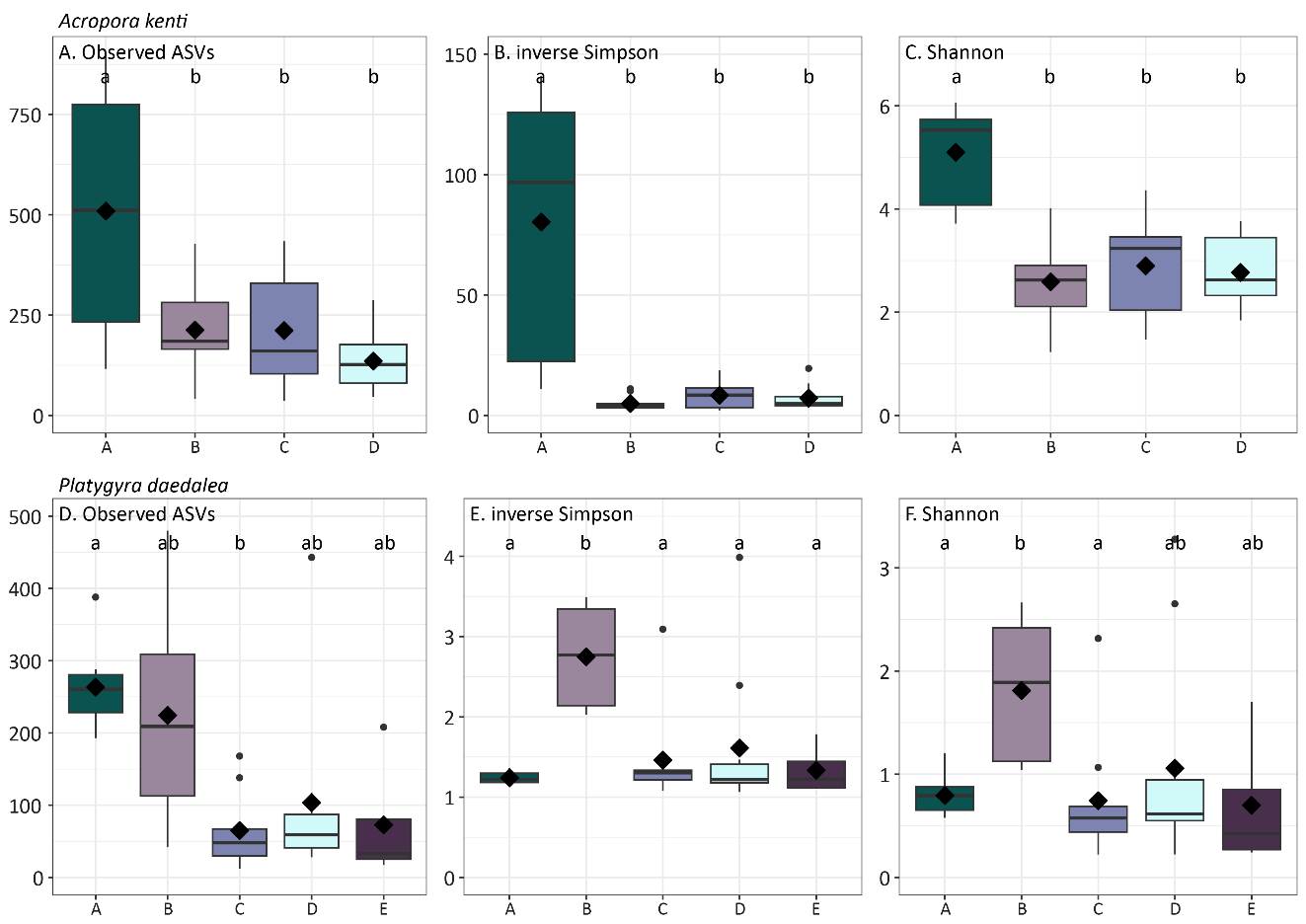


Figure S3: Alpha diversity indices, observed ASVs (A,D), inverse Simpson’s index (B,E), and Shannon’s index (C,F) by coral genotype for A. kenti (A-C), and P. daedalea (D-F). Boxes cover the interquartile range (IQR) and the diamond inside the box denotes the median. Whiskers represent the lowest and highest values within 1.5 × IQR. Different small letters indicate significant differences in Tukey’s honest significant difference (HSD) post hoc tests.


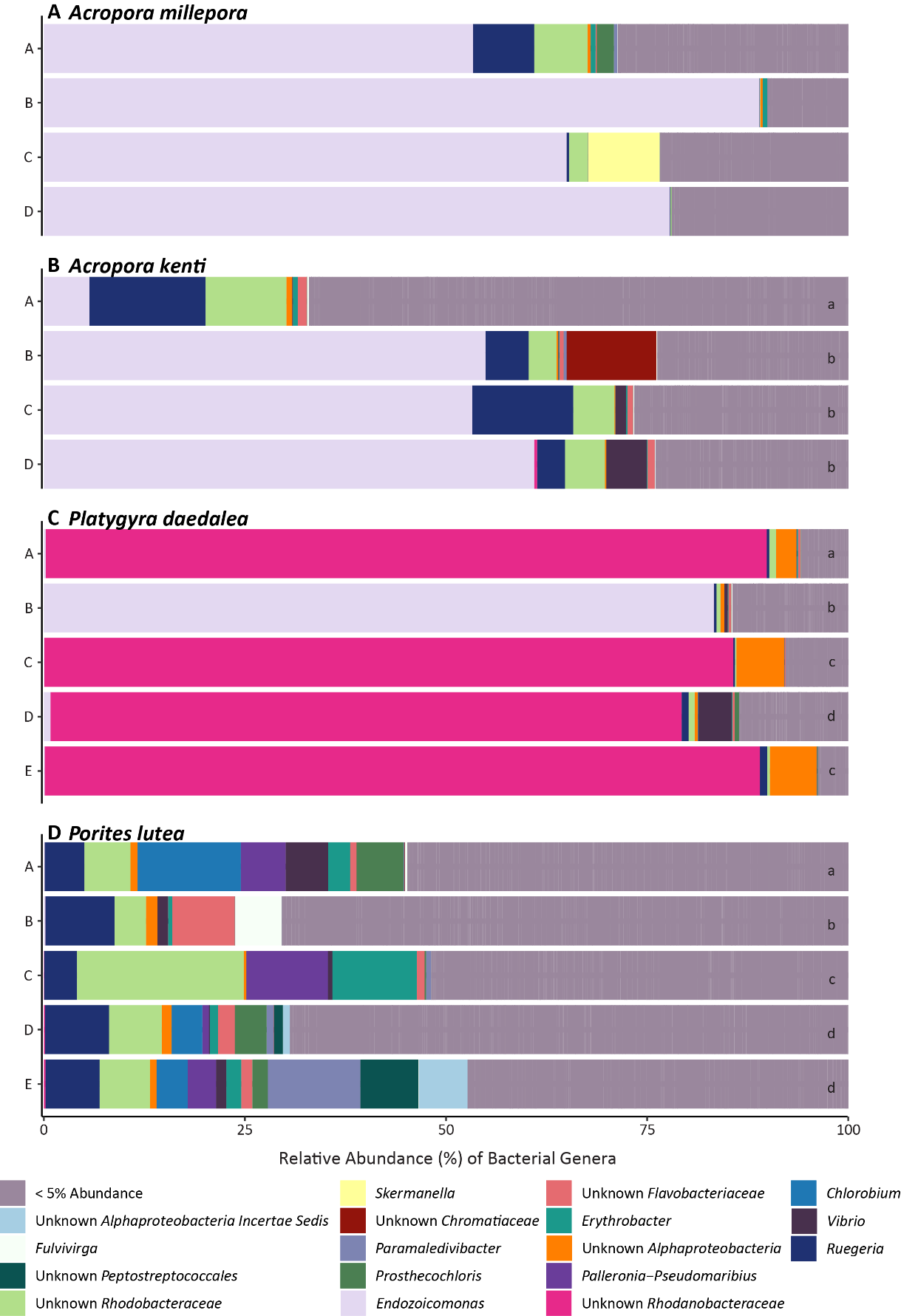


Figure S4: Relative abundance of bacterial genera for *A. millepora* (A), *A. kenti* (B), *P. daedalea* (C), and *P. lutea* (D) by genotype. PMA-treated and untreated samples were pooled as there were no significant differences in community composition for these species. Low abundance genera were pooled into a single category for each species. Where there was a significant difference in bacterial community structure by PERMANOVA, Tukey HSD letters are provided at the right of the barplot. Genotypes within a host species with different lowercase letters indicates that they are significantly different from one another.


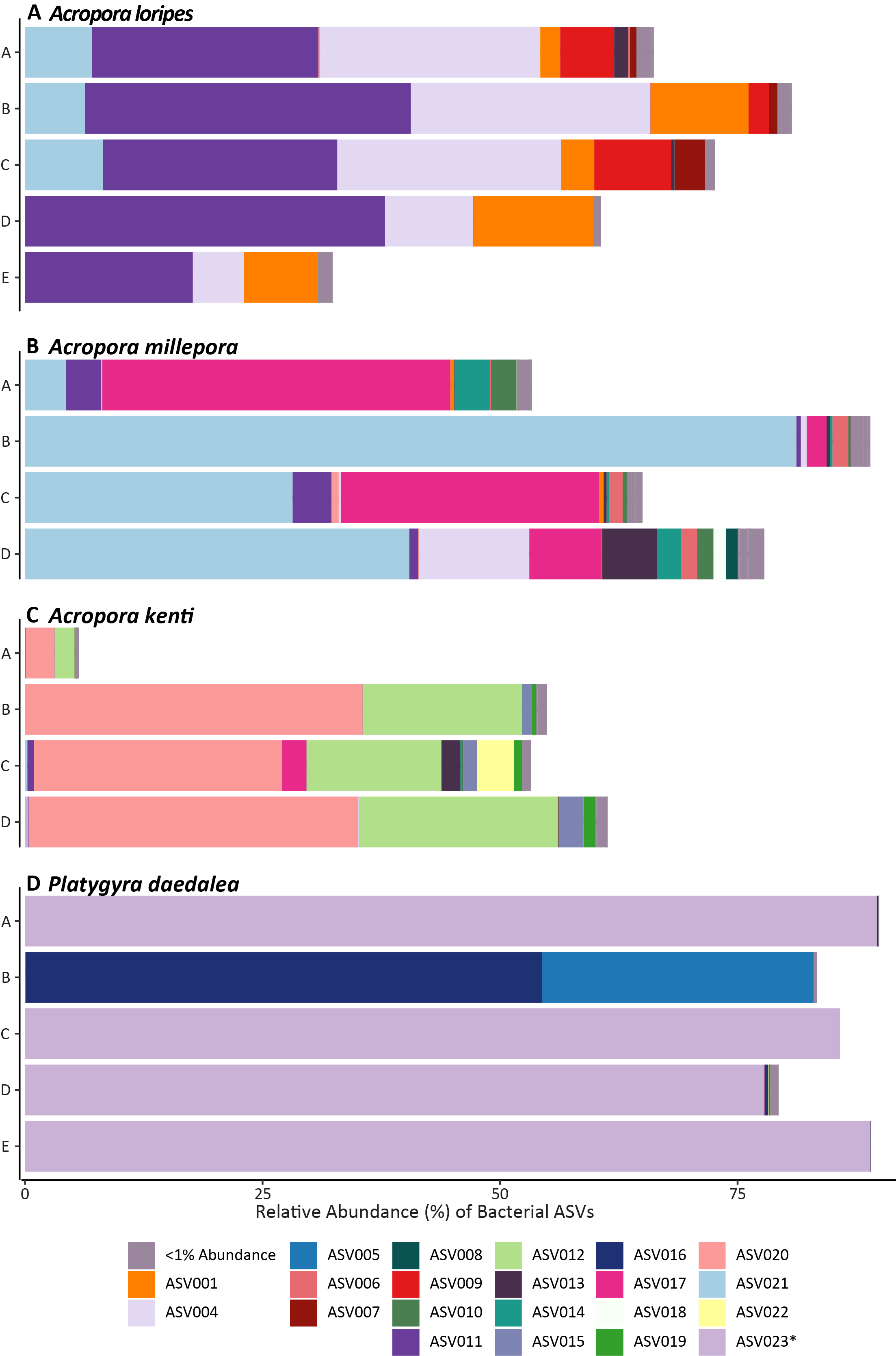


Figure S5: Relative abundance of Endozoicomonas (ASV001, ASV004-22) and unknown Rhodanobacteraceae (ASV023* only) ASVs in A) A. loripes, B) A. millepora, C) A. kenti, and D) P. daedalea by coral genotype. Untreated and PMA-treated samples were pooled for this visualization.
